# Colonic metastasis from cervical carcinoma diagnosed by ligation-assisted endoscopic full-thickness resection

**DOI:** 10.1055/a-2618-2373

**Published:** 2025-09-04

**Authors:** Wen Liu, Sheng Wang, Siyu Sun

**Affiliations:** 1 85024 Department of Gastroenterology, Shengjing Hospital of China Medical University, Shenyang, China

Endoscopic ultrasonography (EUS) plays a crucial role in characterizing subepithelial lesions (SELs) and enabling definitive pathological diagnosis through EUS-guided fine-needle aspiration/biopsy 1 2 3 . Some experts advocate surveillance with EUS for SELs originating from the muscularis propria that are less than 2 cm in diameter 4 . Here, we present a case of colonic metastasis from cervical carcinoma that was successfully diagnosed using ligation-assisted endoscopic full-thickness resection (EFTR) ( Video 1 ).

A colonic metastasis from cervical carcinoma was successfully diagnosed using ligation-assisted endoscopic full-thickness resection.Video 1

A 65-year-old woman with a history of cervical adenocarcinoma, treated surgically 15 months previously, presented with a SEL in the sigmoid colon during colonoscopy ( Fig. 1 ). Miniprobe examination demonstrated a 6 × 4-mm hypoechoic mass originating from the muscularis propria ( Fig. 2 ). Abdominal computed tomography showed no evidence of metastases.

**Fig. 1:**
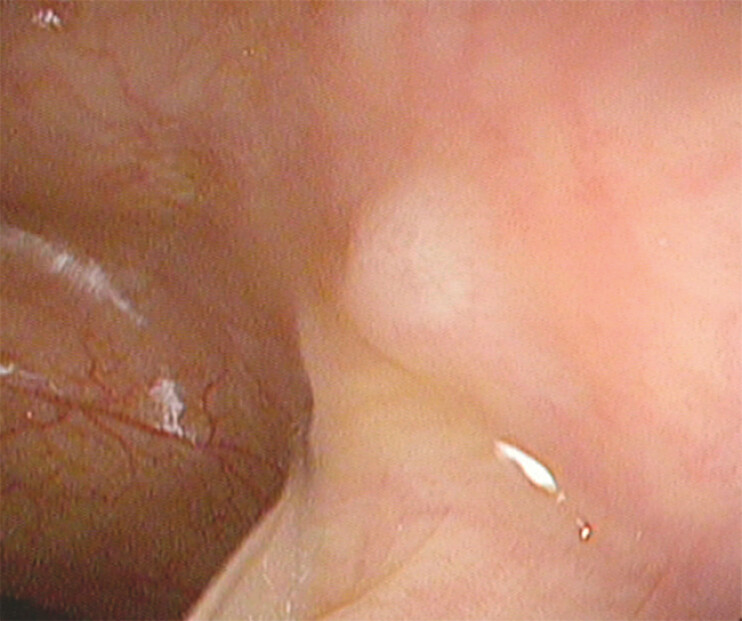
Endoscopic image showing a subepithelial lesion in the sigmoid colon that was detected during colonoscopy.

**Fig. 2:**
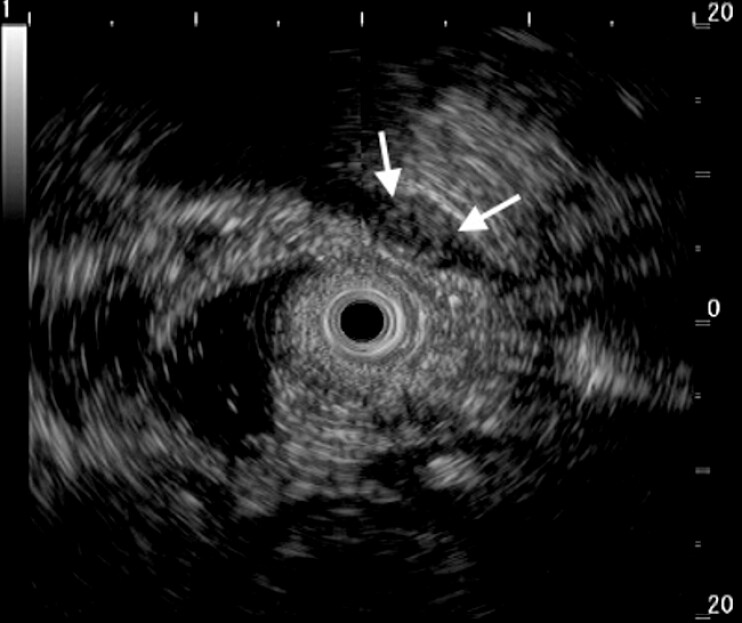
Endoscopic ultrasonography image using a miniprobe showing a 6 × 4-mm hypoechoic mass originating from the muscularis propria.

Given the small size of the lesion and the technical challenges associated with direct endoscopic resection, ligation-assisted EFTR was performed. Firstly, the lesion was marked using a snare. The lesion was then completely suctioned into the ligation device and a rubber band was released. After the mucosa had been incised using a snare to expose the lesion, en bloc full-thickness resection was achieved by snare excision under the rubber band ( Fig. 3 **a** ). Subsequently, the colonic wall defect was closed using metal clips ( Fig. 3 **b** ).

**Fig. 3:**
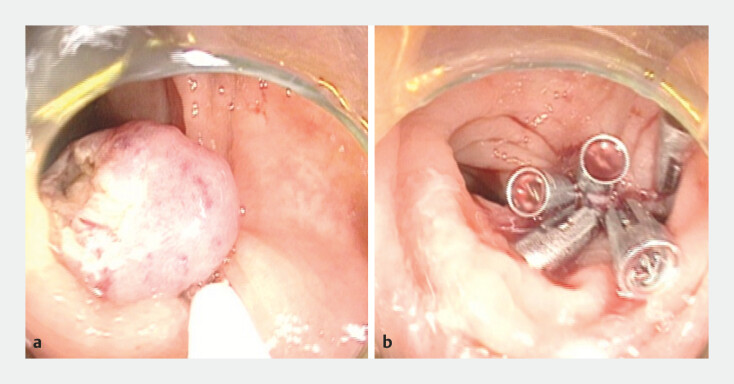
Endoscopic images showing: **a** en bloc full-thickness resection with snare excision under the rubber band after the mucosa had been incised to expose the lesion; **b** the colonic wall defect closed with metal clips.

Histopathological examination revealed adenocarcinoma characterized by irregular tubular structures with infiltrative growth patterns ( Fig. 4 ). Immunohistochemical staining revealed the following profiles: Ki-67 (20%+), P16 (+), PAX8 (focal weak +), SATB2 (−), CK7 (+), and CK20 (−). Based on the morphological features, immunohistochemical profile, and clinical history, the diagnosis was colonic metastasis from cervical carcinoma.

**Fig. 4:**
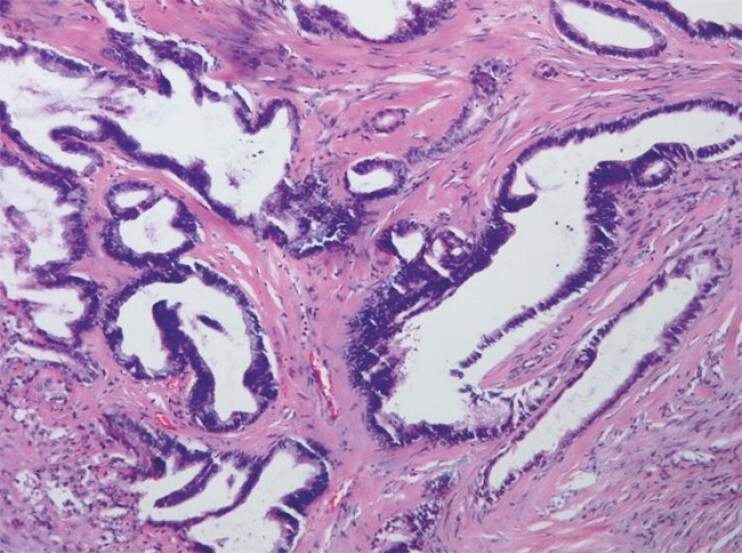
Histopathological image showing adenocarcinoma that is characterized by irregular tubular structures with infiltrative growth patterns.

Our clinical experience suggests that even small lesions carry a potential risk of malignant transformation. It is imperative to achieve en bloc resection while obtaining pathological diagnosis through a safe, effective, and minimally invasive method, particularly for patients with previous malignancy.

Endoscopy_UCTN_Code_TTT_1AQ_2AD_3AF

## Notes

Endoscopy E-Videos https://eref.thieme.de/e-videos
*E-Videos* is an open access online section of the journal *Endoscopy* , reporting on interesting cases and new techniques in gastroenterological endoscopy. All papers include a high-quality video and are published with a Creative Commons CC-BY license. Endoscopy E-Videos qualify for HINARI discounts and waivers and eligibility is automatically checked during the submission process. We grant 100% waivers to articles whose corresponding authors are based in Group A countries and 50% waivers to those who are based in Group B countries as classified by Research4Life (see: https://www.research4life.org/access/eligibility/ ). This section has its own submission website at https://mc.manuscriptcentral.com/e-videos .
